# KAT2A-mediated AR translocation into nucleus promotes abiraterone-resistance in castration-resistant prostate cancer

**DOI:** 10.1038/s41419-021-04077-w

**Published:** 2021-08-12

**Authors:** Dingheng Lu, Yarong Song, Ying Yu, Decai Wang, Bing Liu, Liang Chen, Xuexiang Li, Yunxue Li, Lulin Cheng, Fang Lv, Pu Zhang, Yifei Xing

**Affiliations:** 1 Department of Urology, Union Hospital, Tongji Medical College, Huazhong University of Science and Technology, Wuhan, 430022 China; 2 Department of Emergency Surgery, Union Hospital, Tongji Medical College, Huazhong University of Science and Technology, Wuhan, 430022 China

**Keywords:** Cancer therapeutic resistance, Prostate cancer

## Abstract

Abiraterone, a novel androgen synthesis inhibitor, has been approved for castration-resistant prostate cancer (CRPC) treatment. However, most patients eventually acquire resistance to this agent, and the underlying mechanisms related to this resistance remain largely unelucidated. Lysine acetyltransferase 2 A (KAT2A) has been reported to enhance transcriptional activity for certain histone or non-histone proteins through the acetylation and post-translational modification of the androgen receptor (AR). Therefore, we hypothesised that KAT2A might play a critical role in the resistance of prostate tumours to hormonal treatment. In this study, we found that KAT2A expression was increased in abiraterone-resistant prostate cancer C4-2 cells (C4-2-AbiR). Consistently, elevated expression of KAT2A was observed in patients with prostate cancer exhibiting high-grade disease or biochemical recurrence following radical prostatectomy, as well as in those with poor clinical survival outcomes. Moreover, KAT2A knockdown partially re-sensitised C4-2-AbiR cells to abiraterone, whereas KAT2A overexpression promoted abiraterone resistance in parental C4-2 cells. Consistent with this finding, KAT2A knockdown rescued abiraterone sensitivity and inhibited the proliferation of C4-2-AbiR cells in a mouse model. Mechanistically, KAT2A directly acetylated the hinge region of the AR, and induced AR translocation from the cytoplasm to the nucleus, resulting in increased transcriptional activity of the AR-targeted gene prostate specific antigen (PSA) leading to resistance to the inhibitory effect of abiraterone on proliferation. Taken together, our findings demonstrate a substantial role for KAT2A in the regulation of post-translational modifications in AR affecting CRPC development, suggesting that targeting KAT2A might be a potential strategy for CRPC treatment.

## Introduction

Prostate cancer (PC) is the second most commonly diagnosed malignancy in men worldwide [1, 2]. Since the growth of prostate tumours is highly dependent on androgen, androgen deprivation therapy (ADT) has been considered the primary treatment for locally advanced and metastatic PC for several decades [3]. However, PC tends to progress from hormone-sensitive prostate cancer (HSPC) to castration-resistant prostate cancer (CRPC) after 1–2 years of ADT treatment [4].

Intra-tumour and extra-gonadal androgens are responsible for persistent androgen receptor (AR)-mediated growth in CRPC and are thus considered potential therapeutic targets [5]. Recently, the androgen synthesis inhibitor abiraterone and the AR antagonists enzalutamide/apalutamide were approved as novel hormonal treatments for CRPC, as well as for metastatic HSPC [6–8]. Unfortunately, a certain proportion of PC patients are prone to develop primary and acquired resistance to these new endocrine therapies, including abiraterone, resulting in a gain of less than 6 months in prolonged median progression-free survival (PFS) [9]. The underlying mechanisms that have been proposed to trigger resistance to these next-generation agents include AR mutation and amplification, AR splice variants, post-translational modification (PTM) of AR, alteration of the AR coactivator, cross-talk among pathways, and glucocorticoid receptor overexpression, among others [9–11]. Several studies have reported the mechanisms responsible for enzalutamide resistance [12–14]; however, only a few studies focused on elucidation of the mechanisms responsible for abiraterone resistance. Therefore, the aim of this study was to explore the molecular mechanisms underlying abiraterone resistance, which could suggest new targets for an improved therapeutic outcome.

The PTM of AR plays an important role in the resistance to hormonal treatment and the development of CRPC, including phosphorylation, acetylation, methylation, ubiquitination, and ubiquitin-like modifier (SUMO)-ylation, among others [15, 16]. Acetylation and deacetylation can be regulated by histone acetyltransferases (HATs) and histone deacetylases (HDACs) [17]. Several HATs and HDACs have been found to acetylate/deacetylate non-histone proteins, including AR, and consequently modify cell function. The hinge region of the AR protein, which plays a role in AR localisation from the cytoplasm to the nucleus, is the main site of acetylation, targeting the lysine residues K630, K632, and K633 [18, 19]. Steroid receptor coactivator-1 (SRC1), p300, and p300/cAMP-response element binding protein association factor (p/CAF) can mediate the acetylation of AR, thereby increasing AR transcriptional activity directly or indirectly [18, 20, 21]. In the androgen-sensitive prostate cancer cell line LNCaP, Tip60 knockdown causes localisation of AR in the cytoplasm, which subsequently decreases PSA expression [19]. Arrest defective-1 protein (ARD1) is another HAT that acetylates AR, which tends to promote proliferation in PC cells [22]. Inhibition of histone deacetylases (HDACs), such as HDAC1 [23] and Sirtuin 1 (SIRT1) [24–26], can also result in AR acetylation.

Lysine acetyltransferase 2 A (KAT2A), a member of the HAT family, is located on chromosome 17q21.2 and is associated with enhancement of transcriptional activity through histone acetylation [27–29], histone succinylation, and recruitment of transcriptional co-activators [30]. KAT2A can also acetylate non-histone proteins, such as CCAAT enhancer binding protein beta (CEBPB) [27], polo-like kinase 4 (PLK4) [31] and T-box transcription factor 5 (TBX5) [32], and is involved in destabilising nucleosomes via histone glutarylation [33]. KAT2A has also been shown to play a critical role in carcinogenesis and the progression of several cancers [30, 34, 35]; however, there are no reports of the impact of KAT2A on the non-histone acetylation of AR, and its potential role in primary or secondary resistance to abiraterone.

To evaluate the role of KAT2A in the resistance of PC to abiraterone, in this study, we established a stable C4-2 cell line that was resistant to abiraterone acetate (C4-2-AbiR); this cell line is distinct from the C4-2B cell line, which is resistant to enzalutamide and also showed cross-resistance to abiraterone [36, 37]. We confirmed that the KAT2A expression level was higher in C4-2-AbiR cells than in the parental C4-2 cell line and that KAT2A upregulation was correlated with a worse clinical survival outcome in patients with PC. More specifically, we found that KAT2A directly acetylates the hinge region of AR, thereby inducing AR translocation from the cytoplasm to the nucleus, resulting in increased transcription of the AR-targeted gene, and ultimately preventing abiraterone from inhibiting the proliferation of PC cells. Our results highlight a novel role of KAT2A in CRPC biology, thereby providing further insights for the development of therapeutic strategies that can overcome the major clinical challenge of abiraterone resistance.

## Materials and methods

### Patient tissue specimens and cell lines

A total of 87 sets of specimens of PC tissues and their adjacent normal prostate tissues were obtained from patients who underwent radical prostatectomy for prostate carcinoma at the Department of Urology of Union Hospital affiliated of Tongji Medical College between 2015 and 2019. The intraepithelial neoplasia samples would be excluded from tumour samples. We have acquired the approval from the Institutional Review Board of Tongji Medical College of Huazhong University of Science and Technology before we collected the samples. Consent was obtained for experimentation with human subjects. All specimens were classified according to the 2004 World Health Organization Consensus Classification and Staging System for prostate neoplasms. C4-2 and LNCaP were provided from Prof. Xiaoping Zhang and Prof. Jun Zhao (Union Hospital, Wuhan, China). PC-3 and 293 T were obtained from Shanghai Cell Bank, Chinese Academy of Sciences (Shanghai, China). Cells were maintained in RPMI 1640 medium (Hyclone, GE Healthcare Life Sciences, Logan, UT, USA) with 10% foetal bovine serum (Biologic Industries, Kibbutz Beit Haemek, Israel) that contained 1% penicillin/streptomycin (Beyotime Institute of Biotechnology, Nanjing, China) at 37 °C in 5% CO_2_ and 95% humidified air.

### Establishment of the abiraterone-resistant cell line C4-2-AbiR

The starting treatment concentration of abiraterone acetate (MedChemExpress, NJ 08852, USA) in the parental C4-2 cell culture medium was 0.5 μM. At this concentration, the cells were stably passaged three times. The drug concentration was then increased, and the culture was continued. The concentration gradients of abiraterone acetate were 1 μM, 2 μM, 4 μM, 8 μM, 10 μM, 14 μM, 16 μM, 20 μM, 22 μM and 25 μM. Cells were maintained in phenol red-free RPMI 1640 medium (Tianjin Hao Yang Biological Manufacture CO., LTD, Tianjin, China) with 10% charcoal stripped fetal bovine serum (Biologic Industries, Kibbutz Beit Haemek, Israel) that contained 1% penicillin/streptomycin (Beyotime Institute of Biotechnology, Nanjing, China) at 37 °C in 5% CO_2_ and 95% humidified air. After 6 months of induction, the stable abiraterone-resistant cell line was obtained and named C4-2-AbiR.

### Detection of cell cycle

Cell cycle detection were performed by flow cytometer (Beckman Coulter, Indianapolis, IN, USA). Cells were harvested by centrifuging at 1500 rpm for 5 min, fixed with ethanol at 4 °C overnight, and stained with PI. The percentage of cells in each cell cycle phase was analyzed.

### Colony formation

C4-2-AbiR, C4-2 and LNCaP cells transfected with the plasmids were cultured in 6-wells plates at the density of 800–1000 cells per well. Plates were incubated at 37 °C in 5% CO_2_ for 2-3 weeks, and the colonies with more than 50 cells were scored. The cell colonies were immobilized with 4% paraformaldehyde and dyed by crystal violet. When the counting of plates has been finished, we used formulation to calculate the rate of colony formation. (Rate = the number of colonies/ the number of seeded cells).

### Quantitative real-time PCR

cDNA was synthesized from total RNA by using the iScript cDNA synthesis kit (Bio-Rad, Hercules, CA, USA) following manufacturer protocols. Quantitative real-time PCR was performed by using the ABI Power SYBR Green PCR Master Mix (Applied Biosystems, Foster City, CA, USA) with the 7900 HT Sequence Detection System (Applied Biosystems). Glyceraldehyde 3-phosphate dehydrogenase was used as internal control. PCR primer pairs were synthesized by Sangon Biotech (Shanghai, China), and primer sequences are presented in Supplementary Table 1.

### Western blotting

Total protein was isolated by using a RIPA lysis buffer (Beyotime Institute of Biotechnology), separated on SDS-PAGE gels, and transferred to PVDF membranes (EMD Millipore, Billerica, MA, USA). Membranes were blocked by 5% nonfat milk in Tris-buffered saline with Tween-20, then incubated with primary Abs overnight at 4 °C and with corresponding secondary Abs (1:4000; Catalogue No: SA00001-1 and SA00001-2; ProteinTech, Chicago, IL, USA). The following primary Abs were used: rabbit anti-human KAT2A (1:1000; Catalogue No: 3305 T; Cell Signaling Technology, Danvers, MA, USA), rabbit anti-human AR (1:1000; Catalogue No: 5153 T; Cell Signaling Technology), rabbit anti-human PSA (1:1000; Catalogue No: 10679-1-AP; ProteinTech), rabbit anti-human acetylated lysine (1:1000; Catalogue No: 9441 S; Cell Signaling Technology), rabbit anti-human His tag (1:500; Catalogue No: 10001-0-AP; ProteinTech), rabbit anti-human Flag tag (1:1000; Catalogue No: 20543-1-AP; ProteinTech). Mouse anti-human β-actin (1:5000; Catalogue No: 66009-1-Ig; ProteinTech) and rabbit anti-human lamin B1 (1:2000; Catalogue No: 12987-1-AP; ProteinTech) were used as loading controls. Protein bands were visualized with ECL (Beyotime Institute of Biotechnology).

### Immunohistochemistry

Samples included those from 87 patients with PC and xenograft tumours in vivo. Samples were paraffinized, rehydrated, and blocked with 3% of H_2_O_2_, followed by incubation with normal goat serum (Vector Laboratories, Burlingame, CA, USA). After incubation with the primary antibody overnight at 4 °C, sections were washed with PBS and incubated with the biotinylated secondary antibody (Vector Laboratories), followed by incubation with Vectastain ABC Reagent (Vector Laboratories). The visualization signals were developed using diaminobenzidine (DAB, Vector Laboratories), and the slides were counterstained with hematoxylin. The images were captured using an Olympus BX60 microscope (Olympus, Japan). Immunoreactivity was scored based on a combination of both the percentage and intensity of positively stained tumour cells to generate an H-score. Staining intensity was divided into four categories as follows: no staining, 0; weak staining, 1; moderate staining, 2; strong staining, 3. H-score was determined according to the formula: (% of weak staining×1) + (% of moderate staining×2) + (% of strong staining×3), yielding a range from 0 to 300. Samples from patients were stained with KAT2A and AR and those from xenograft tumours were stained with KAT2A, AR, PSA and Ki67.

### Cell viability assay

Control group and treatment group cells were plated on 96-well plates at 5000 cells/well and cultured with complete medium overnight. After 24 h, 10 ml of Cell Counting Kit- 8 solution (Dojindo Laboratories, Kumamoto, Japan) was added to each well and the plate was incubated for 2 h at 37 °C. This process was repeated for four days. Absorbance at 450 nm was measured on a microplate reader (Tecan, Mannedorf, Switzerland).

### Plasmids transfection and lentiviral constructs

We constructed seven types of plasmids which are plasmids overexpressing KAT2A, plasmids with knocked-down KAT2A, plasmids overexpressing AR, AR-K630A mutant plasmids, AR-K632A mutant plasmids, AR-K633A plasmids, AR-K630Q mutant plasmids (Vigene Biosciences, Shangdong, China). Plasmids and vectors were transfected into C4-2 and LNCaP with Lipofectamine 2000 according to protocol. C4-2 and LNCaP were infected with lentivirus overexpressing KAT2A (Hanheng Biotechnology, Shanghai, China).

### Immunofluorescence staining

Cells were fixed in 4% paraformaldehyde, permeated by 0.3% Triton X-100, and blocked with 3% BSA for 1 h at 37°C, followed by incubation with primary KAT2A and AR antibodies. All data were analyzed via Nikon A1Si Laser Scanning Confocal Microscope (Nikon Instruments Inc., Japan).

### Co-immunoprecipitation and immunoprecipitaion

Subconfluent proliferating cells in 100-cm^2^ dishes were harvested, collected in lysis buffer, left on ice for 30 min, sonicated, and centrifuged at 15000 rpm for 15 min at 4 °C. Supernatants were collected. Each immunoprecipitation (IP) was carried out using 5-μg antibody and 500-μg protein. The precipitated proteins were collected using protein A + G beads, washed, eluted in boiling Laemmli sample buffer, and subjected to Western blotting. Briefly, 100-μg protein from each group was fractionated on 10% SDS-polyacrylamide gels and transferred to nitrocellulose membranes (Millipore, Bedford, MA, USA). The membranes were then blotted with primary antibodies, followed by the secondary antibody and developed with enhanced chemiluminescence reagent (Beyotime Institute of Biotechnology). The primary antibodies used in this study were obtained from (ProteinTech, Chicago, IL, USA) and (Cell Signaling Technology, Danvers, MA, USA).

### Luciferase reporter assays

PC-3 cells were seeded in 24-well plate (6 × 10^4^cells per well) 24 h before transfection. The pGL3-basic plasmids containing promoter of PSA and renilla luciferase reporter vectors (pRL-TK) were co-transfected with AR and KAT2A overexpression plasmids, to determine the combination level of AR and promoter of PSA. Besides, plasmids of the transcription binding sites of KAT2A (JASPAR database) were co-transfected with AR overexpression plasmids to determine the specific binding sites of KAT2A and AR. 48 hours after transfection, the firefly and renilla luciferase activities were measured with Dual-Luciferase® Reporter Assay System (Promega, USA) as described in specification.

### Tumour xenografts

Three- to four-week-old castrated male nude mice were purchased from Beijing Vital River Laboratory Animal Technology Co., Ltd. Their care was in accordance with institution guidelines. Approximately 5×10^6^ C4-2-AbiR cells with knockdown of KAT2A or control cells suspended in 100 μl of serum-free medium were injected subcutaneously into the armpit on the right side of the mice. Tumour volumes (V) were measured every five days based on measurements of length (L) and width (W) and calculated as V = (L×W^2^)/2. When the tumours reached 100–200 mm^3^, the mice were randomized equally, treated with vehicle or abiraterone acetate (60 mg/kg) and prednisolone (0.1 mg/kg) twice a week, and sacrificed ~40 days later (*n* = 5 each group). Mice that died before completion of experimental protocol were excluded from further data analysis. Investigators who performed endpoint analyses were blinded to group allocation.

### Bio-informatics research

Data of GDS2545 in Gene Expression Omnibus (GEO) database were used (Adjacent group: 63, Tumour group: 65). Data from J B Welsh et al in Oncomine database were used [38]. Data of Prostate Adenocarcinoma Group (PRAD group) from TCGA database were used.

### Statistical analysis

All statistical analyses were performed using Prism 8.0 (GraphPad) and SPSS 22.0 (IBM Corporation). All in vitro experiments were repeated three times. All data are presented as the mean ± SD. Survival information was verified by Kaplan–Meier analysis and compared using the log-rank test. A two-tailed unpaired Student’s t test was used to determine the *p* values, which were considered significant at less than 0.05. Test of normal distribution would be performed to decide either parametric test or nonparametric test to be used. F test would be performed to compare variances between the groups to decide whether Welch’s correction should be used.

## Results

### KAT2A is up-regulated in PC tissues and is correlated with worse prognosis

**Fig. 1:**
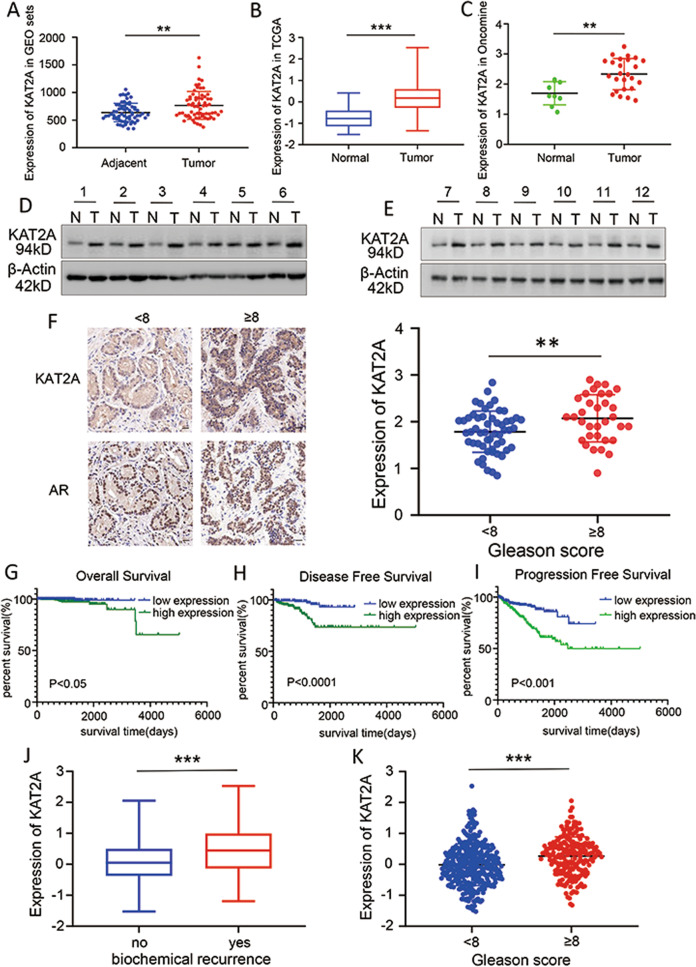
KAT2A is up-regulated in PC tissues and is correlated with worse prognosis. **A–C** The expression levels of KAT2A in adjacent/normal and tumour were searched in GEO, TCGA, Oncomine respectively. **D, E** Specimens were stratified into “GS < 8 group” and “GS ≥ 8 group” according to Gleason score (GS), followed by random selection of 6 samples from each group for subsequent western blot experiments. (N: normal, T: tumour). **F** The correlation between KAT2A, AR expression level and Gleason score were identified by IHC with 54 patients in the “GS < 8 group” and 33 patients in “GS ≥ 8 group”. **G–I** OS, DFS, PFS were analysed according to high or low expression of KAT2A and log-rank *t* test was used. **J** The correlation of KAT2A expression and biochemical recurrence. **K** The correlation of KAT2A expression and Gleason score. *p < 0.05, **p < 0.01, ***p < 0.001.

We mined the GEO database and found that KAT2A expression was up-regulated in PC tumours compared to levels in the adjacent tissues (Fig. 1A) [39–42]. Similar results were found based on information obtained from TCGA and Oncomine databases (Fig. 1B–C) [38, 43]. Consistently, the protein level of KAT2A was higher in tumour tissues than in the adjacent tissues collected from 12 patients with PC (Fig. 1D, E). Moreover, IHC with specific antibodies showed that the expression of KAT2A was higher in tissues from patients with high-risk PC (Fig. 1F). According to TCGA data, patients with high KAT2A expression had worse overall survival (*p* < 0.05), disease-free survival (*p* < 0.0001), and PFS (*p* < 0.001) than those with low KAT2A expression (Fig. 1G–I). Patients with a definite biochemical relapse or Gleason score ≥8 showed higher expression of KAT2A (Fig. 1J–K) compared to that in patients with no biochemical relapse or a Gleason score <8. Taken together, these results implied that KAT2A is up-regulated in PC and has a strong correlation with a worse prognosis.

### KAT2A is up-regulated in the newly established C4-2-AbiR cell line

**Fig. 2:**
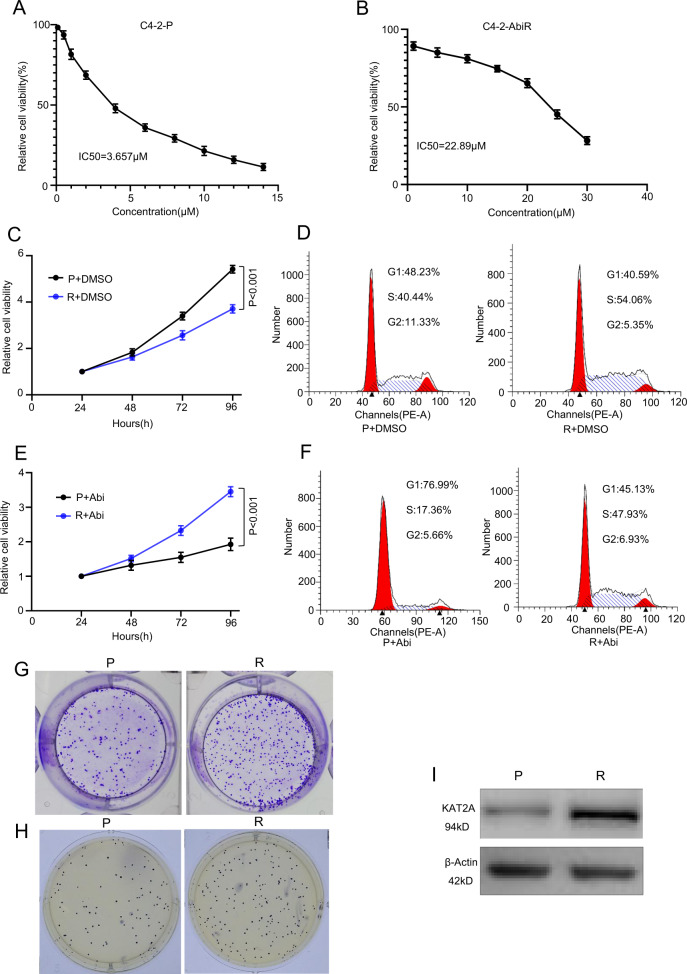
KAT2A is up-regulated in the newly established C4-2-AbiR cell line. **A, B** IC50 of C4-2-P and C4-2-AbiR tested by CCK-8. **C, E** Relative cell viability of C4-2-P and C4-2-AbiR with or without abiraterone detected by CCK-8. **D, F** Cell cycle of C4-2-P and C4-2-AbiR with or without abiraterone detected by flow cytometry. **G, H** Proliferation of C4-2-P and C4-2-AbiR showed by colony formation and soft agar assay. **I** The protein expression of KAT2A in C4-2-P and C4-2-AbiR.

The IC_50_ values of both the parental C4-2 (C4-2-P) and C4-2-AbiR cell lines are shown in Fig. 2A and B. The CCK-8 assay showed that C4-2-AbiR cells proliferated more slowly than C4-2-P cells (Fig. 2C). Flow cytometry results showed that C4-2-AbiR cells were blocked in the S stage (Fig. 2D), which was consistent with the CCK-8 assay results. After treatment with abiraterone acetate, C4-2-P cells were blocked in the G1 stage compared to that in the control cells treated with DMSO (Fig. 2F), indicating that the relative cell viability of C4-2-P cells was repressed, whereas no significant effect of abiraterone acetate on C4-2-AbiR proliferation was detected via the CCK-8 assay (Fig. 2E). Colony formation and soft agar assays further indicated that C4-2-AbiR cells could proliferate even when exposed to the IC50 of abiraterone acetate, whereas the growth of C4-2-P cells was suppressed at the same abiraterone acetate concentration (Fig. 2G, H). Considering that these results suggested that KAT2A plays a critical role in PC, we also determined KAT2A expression levels in these two cell lines and found that KAT2A expression was up-regulated in C4-2-AbiR cells compared with that in C4-2-P cells (Fig. 2I). These results confirmed that KAT2A plays a role in the resistance of PC cells to abiraterone.

### The regulation of KAT2A can modify resistance to abiraterone in PC

**Fig. 3:**
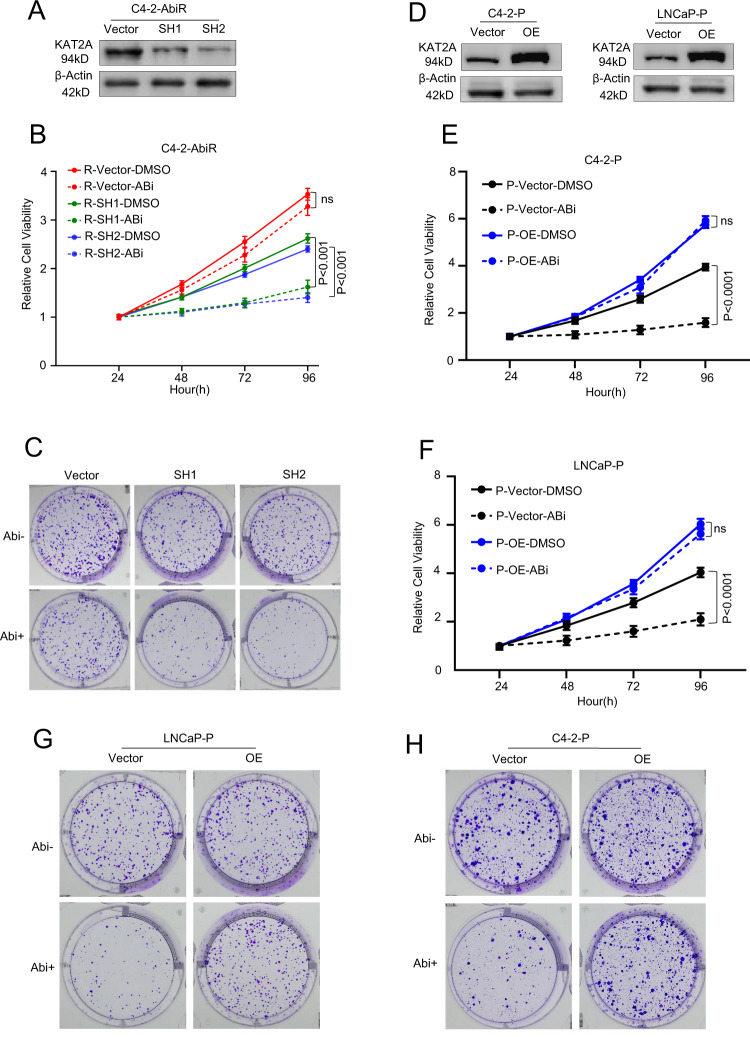
The regulation of KAT2A can modify resistance to abiraterone in PC. **A** Identification of KAT2A silence in C4-2-AbiR. **B** Relative cell viabilities of C4-2-AbiR stably transfected with sh-KAT2A plasmids or vectors were detected by CCK-8. **C** Proliferation ability of C4-2-AbiR stably transfected with sh-KAT2A plasmids or vectors were detected by colony formation assay. **D** Identification of KAT2A overexpression in C4-2-P and LNCaP-P. **E, F** Relative cell viabilities of C4-2-P and LNCaP-P stably transfected with OE-KAT2A plasmids or vectors were detected by CCK-8. **G, H** Proliferation ability of C4-2-P and LNCaP-P stably transfected with OE-KAT2A plasmids or vectors were detected by colony formation assay.

To explore the function of KAT2A in PC abiraterone resistance, we knocked it down in C4-2-AbiR cells using KAT2A-specific shRNA (Fig. 3A). Although abiraterone had no significant effect on the proliferation of C4-2-AbiR cells, proliferation of these cells was inhibited by abiraterone acetate treatment after knocking down KAT2A (Fig. 3B). The colony formation assay revealed that knocking down KAT2A reduced the colony formation rate in the presence of abiraterone acetate (Fig. 3C). To further confirm the role of KAT2A in abiraterone resistance, KAT2A was overexpressed in parental LNCaP (LNCaP-P) and C4-2-P cells (Fig. 3D). As shown in Fig. 3E and F, abiraterone could significantly inhibit the viability of LNCaP-P and C4-2-P cells, which was rescued by the overexpression of KAT2A. The colony formation assay also showed that LNCaP-P and C4-2-P cells with KAT2A overexpression could survive treatment with abiraterone, which was not the case for control cells expressing the empty vector (Fig. 3G–H). Interestingly, KAT2A knockdown in the AR-negative PC cell lines PC-3 and DU145 had no impact on proliferation in the context of abiraterone treatment (Fig S1A, B). Taken together, these results suggest that diminishing KAT2A expression in PC cells might overcome abiraterone resistance, which could prove to be therapeutically beneficial for patients.

### KAT2A promotes AR activity by facilitating its nuclear translocation

**Fig. 4:**
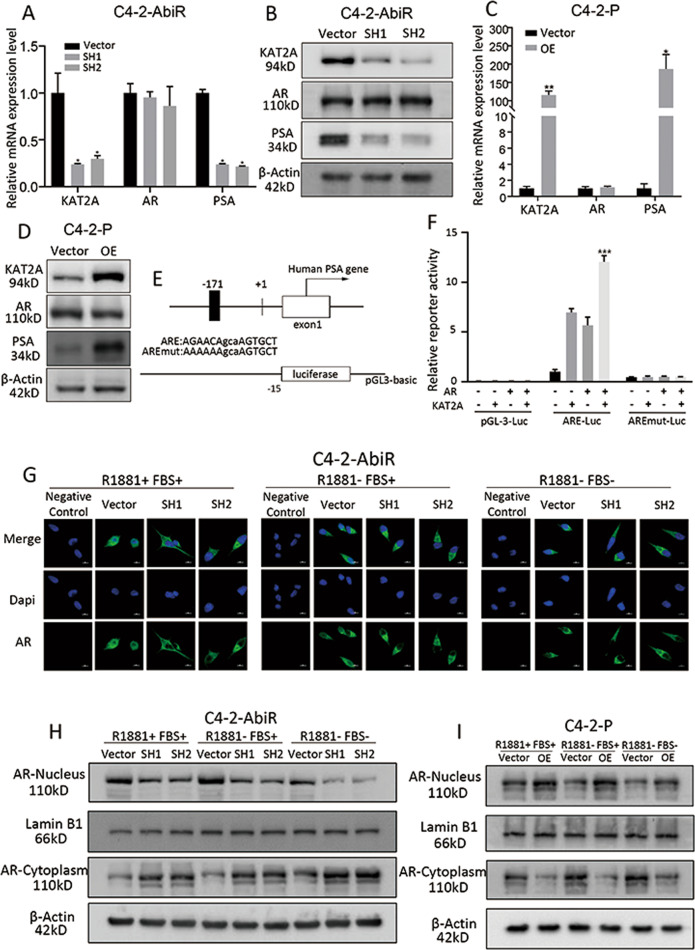
KAT2A promotes AR activity by facilitating its nuclear translocation. **A** Relative mRNA expression levels of KAT2A, AR and PSA in C4-2-AbiR stably transfected with sh-KAT2A or vectors were detected by qRT-PCR. **B** The protein levels of KAT2A, AR and PSA in C4-2-AbiR stably transfected with sh-KAT2A or vectors were detected by western blotting. **c** Relative mRNA expression levels of KAT2A, AR and PSA in C4-2-P stably transfected with OE-KAT2A or vectors were detected by qRT-PCR. **D** The protein levels of KAT2A, AR and PSA in C4-2-P stably transfected with OE-KAT2A or vectors were detected by western blotting. **E** Pattern of pGL3-basic containing promoter of PSA and mutant. **F** Dual-luciferase assays performed using the control pGL3, ARE, and AREmut constructs in the presence or absence of exogenous KAT2A and AR. **G** Location of AR in C4-2-AbiR stably transfected with sh-KAT2A or vectors was detected by IF. **H** Aggregate of AR in cytoplasm or nucleus in C4-2-AbiR stably transfected with sh-KAT2A or vectors were extracted and detected. **I** Aggregate of AR in cytoplasm or nucleus in C4-2-P stably transfected with oe-KAT2A or vectors were extracted and detected. *p < 0.05, **p < 0.01, ***p < 0.001.

AR is a key transcription factor that plays a vital role during PC progression, as well as in endocrine therapy resistance. Considering that high KAT2A expression was associated with abiraterone resistance, to explore the underlying mechanism, we next evaluated whether KAT2A induced resistance through the AR signalling pathway. First, we knocked down KAT2A expression and examined protein and mRNA levels of AR and the AR-targeted gene PSA in C4-2-AbiR cells. As shown in Fig. 4A–B, knockdown of KAT2A down-regulated PSA expression but did not impact AR expression. In addition, KAT2A overexpression in C4-2-P and LNCaP-P cells enhanced both the protein and mRNA levels of PSA, whereas AR expression was unchanged (Fig. 4C, D, S3A, S3B). In line with these findings, there was no correlation between KAT2A and AR expression levels (Fig. S2A and S2B). IHC also showed that there was no obvious change in AR expression between “Gleason Score <8” group and “Gleason Score ≥ 8” groups (Fig. 1F).

To further evaluate whether the AR signalling pathway is involved in the KAT2A-mediated resistance to abiraterone, we investigated the transcriptional function of AR by designing dual-luciferase reporter plasmids containing wild-type and mutant PSA promoters (Fig. 4E) and co-transfected them into PC-3 cells. KAT2A overexpression increased the relative reporter activity compared to that in the empty vector control group. However, up-regulation of KAT2A failed to achieve the same effects when co-transfected with the mutant PSA promoter (Fig. 4F). Considering that AR should be activated by its translocation from the cytoplasm to the nucleus, we subsequently detected AR subcellular localisation according to KAT2A expression status. Cell immunofluorescence showed that when KAT2A was knocked down, AR was mainly distributed in the cytoplasm (Fig. 4G). In contrast, up-regulating KAT2A expression drove AR into the nucleus in LNCaP-P and C4-2-P cells (Fig. S3D, S3E). Next, we separated nuclear and cytoplasmic proteins to analyse the expression of AR. KAT2A knockdown in C4-2-AbiR cells decreased the levels of AR protein in the nucleus (Fig. 4H). In contrast, the exogenous upregulation of KAT2A in C4-2-P and LNCaP-P cells drove AR translocation into the nucleus (Fig. 4I, S3C).These data indicated that KAT2A may induce resistance to abiraterone through the AR signalling pathway.

### KAT2A facilitates AR translocation from the cytoplasm to the nucleus by acetylating AR at K630, and AR can increase the expression of KAT2A

**Fig. 5:**
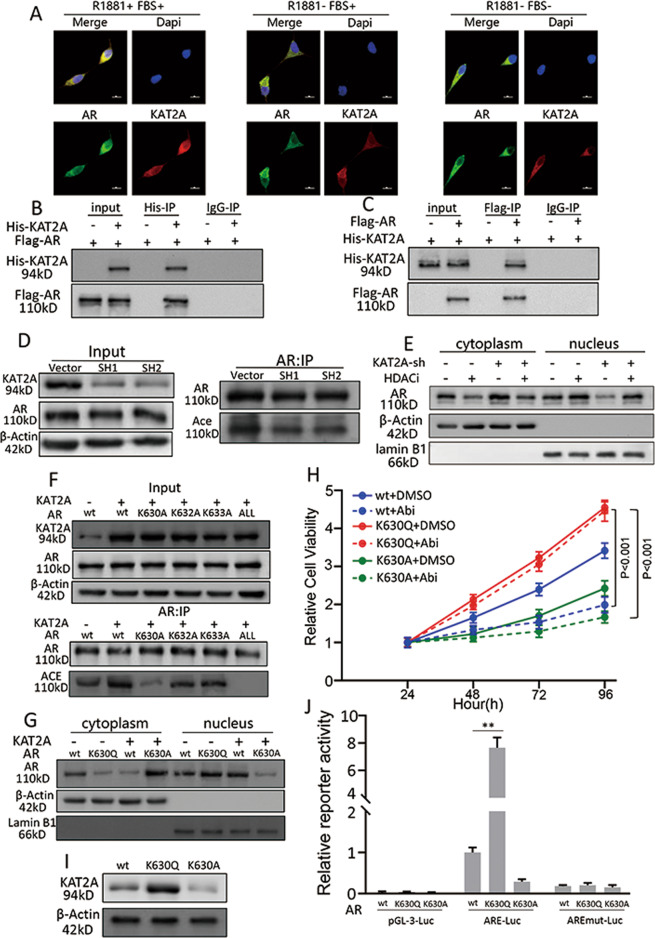
KAT2A facilitates AR translocation from the cytoplasm to the nucleus by acetylating AR at K630, and AR can increase the expression of KAT2A. **A** Demonstrative immunofluorescence images of KAT2A and AR protein localisation in C4-2-AbiR cells. **B, C** PC-3 cells were transiently transfected with His-KAT2A and Flag-AR for 2 days, and whole-cell lysates were immunoprecipitated with anti-His (**B**) or -Flag (**C**) antibodies and blotted with the corresponding antibodies. **D** Acetylation level of AR in C4-2-AbiR stably transfected with sh-KAT2A detected by pan-acetylation antibody after immunoprecipitation by AR antibody from cell lysates. **E** Aggregate of AR in cytoplasm or nucleus in C4-2-AbiR stably transfected with sh-KAT2A or vectors with or without HDAC inhibitor were extracted and detected. **F** AR of wild-type, K630A, K632A, K633A were constructed and co-expressed with oe-KAT2A or vectors in AR negative PC-3 cell line and acetylation of AR were detected after immunoprecipitation by AR antibody. **G** AR in cytoplasm and nucleus were extracted respectively after AR of wild-type (wt), K630Q, K630A were constructed and expressed. **H** Relative cell viability of PC-3 cell line stably transfected with AR of wt, K630Q or K630A with or without abiraterone. **I** Protein level of KAT2A were detected in PC-3 stably transfected with AR of wt, K630Q, K630A. **J** Dual-luciferase assays performed using the control pGL3, ARE, and AREmut constructs in the presence of AR of wt, K630Q, K630A.*p < 0.05, **p < 0.01.

KAT2A can act as an acetyltransferase for both histone and non-histone protein substrates[27, 31, 32]. Considering that the overexpression of KAT2A could promote AR translocation, we hypothesised that KAT2A might facilitate AR translocation via AR acetylation. Immunofluorescence assays showed that KAT2A and AR exhibited notable co-localisation in C4-2-AbiR cells. R1881 and FBS-depleted conditions seemed to have no apparent effect on the co-localisation of KAT2A and AR (Fig. 5A, S4E). Immunoprecipitation using anti-His with subsequent anti-Flag western blot was used to detect cross-linked KAT2A and AR in PC-3 cells, which also showed that KAT2A and AR exhibited an obvious co-localisation (Fig. 5B, C). We then precipitated AR from C4-2-AbiR cells using an anti-AR antibody and tested the levels of acetylation using an anti-acetylation antibody. As shown in Fig. 5D, decreased levels of AR acetylation were observed in the KAT2A shRNA group compared to those in the empty vector control group of C4-2-AbiR cells. In contrast, KAT2A overexpression in parental cell lines resulted in elevated AR acetylation levels (Fig. S4A, B). Moreover, treatment with an HDAC inhibitor rescued the effect of KAT2A knockdown in C4-2-AbiR cells (Fig. 5E).

Mutation of the K630, K632, and K633 residues in the hinge region to alanine has been reported to cause a loss of AR acetylation [15]. To confirm the identity of the residues as possible acetylation targets of KAT2A, plasmids expressing AR proteins with lysine-to-alanine mutations were generated individually and in combination. After immunoprecipitating AR from the cell lysate, we found that the acetylation level of AR was evidently decreased in the acetylation-blocking K630A mutant, whereas a relatively diminished change was observed with K632A and K633A, indicating that K630 is the chief acetylated position targeted by KAT2A (Fig. 5F).

Furthermore, we established plasmids expressing acetylation-deficient AR^K630A^ and the acetylation-mimicking AR^K630Q^ to determine the role of K630 in KAT2A-mediated AR translocation. K630Q mimicked the effect of KAT2A overexpression in PC-3 cells, whereas K630A could offset the effect of KAT2A overexpression (Fig. 5G). Proliferation and cell viability analyses showed that the K630Q mutant could rescue the inhibitory effect of abiraterone (Fig. 5H, S4C). Moreover, we found that KAT2A-SH could not rescue the AR nuclear translocation resulting from AR^K630Q^, and KAT2A-OE could not rescue AR accumulation in the cytoplasm resulting from AR^K630A^ in the colony formation assay and cell growth experiments with abiraterone treatment, which further emphasised the acetylation function of KAT2A targeting AR in abiraterone resistant CRPC (Fig. S4F, S4G). Furthermore, considering the transcriptional activity of AR, we evaluated whether acetylated AR could bind to the KAT2A promoter and facilitate its transcription. Plasmids containing wild-type AR, AR^K630A^, or AR^K630Q^ were transfected into PC-3 cells; we observed that KAT2A expression increased significantly in the K630Q group (Fig. 5I). Thereafter, co-transfection of pGL3-basic plasmids containing the KAT2A promoter (Fig. S4D) with wild-type AR, AR^K630A^, or AR^K630Q^ in PC-3 cells showed that K630Q could upregulate KAT2A expression, whereas K630A decreased KAT2A expression levels (Fig. 5J).

In summary, these results showed that KAT2A could facilitate AR translocation from the cytoplasm to the nucleus via the acetylation of AR at K630, and acetylated AR increased the expression of KAT2A, suggesting the presence of a positive feedback loop.

### Knockdown of KAT2A reduces abiraterone-resistant CRPC proliferation in vivo

**Fig. 6:**
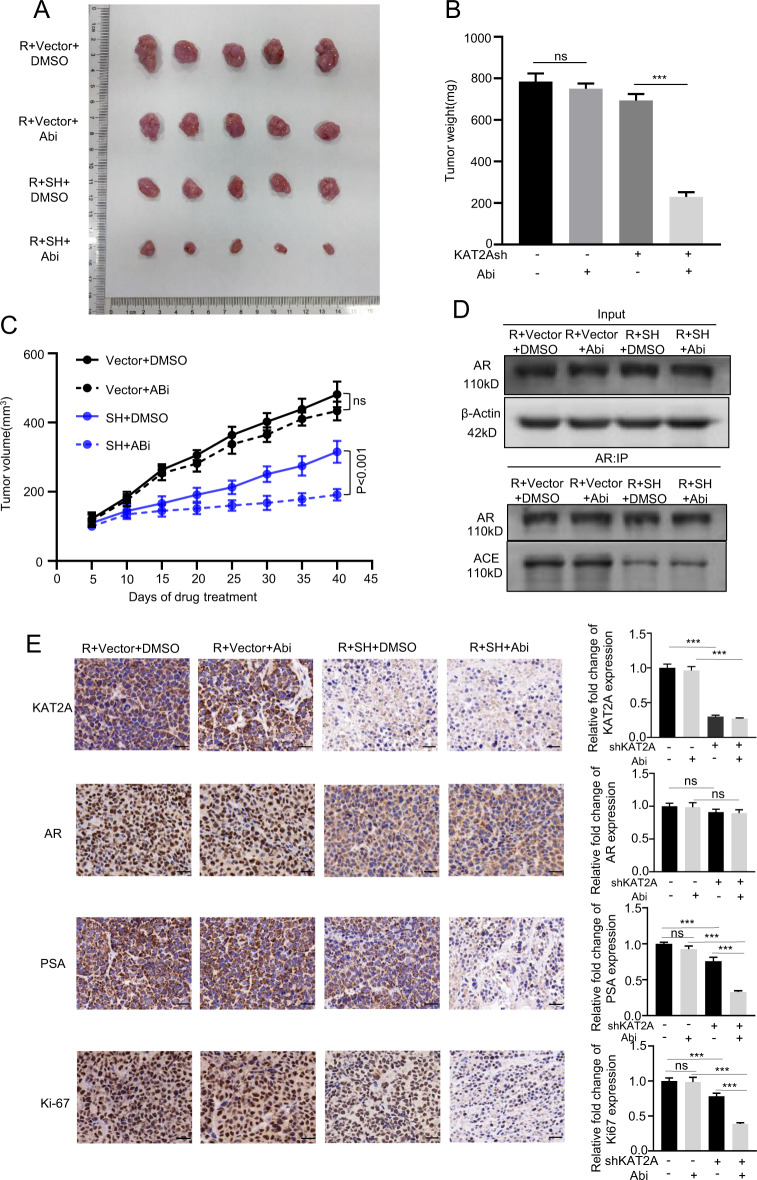
Knockdown of KAT2A reduces abiraterone-resistant CRPC proliferation in vivo. **A** Nude mice bearing C4-2-AbiR with stable KAT2A knockdown xenografts were treated with vector control or abiraterone acetate and prednisolone for ~7 weeks (*n* = 5). **B** Tumours were weighed after resection at the end of the experiment. **C** Tumour volumes were measured every 5 days. Data are shown as the means ± SD. **D** Acetylation of AR in xenograft tissue was detected by western blot. **E** IHC detection of the expression of KAT2A, AR, PSA and Ki-67 in each group was performed. *p < 0.05, **p < 0.01, ***p < 0.001.

The experiments described previously herein demonstrated that KAT2A knockdown partially reversed abiraterone resistance in C4-2-AbiR cells and that KAT2A mediates AR translocation into the nucleus by acetylating K630 in the hinge region of AR. To verify these effects of KAT2A in vivo, we transplanted KAT2A-stably knocked down C4-2-AbiR cells into castrated male BALB/c mice. The mice were fed abiraterone acetate (60 mg/kg) and prednisolone (0.1 mg/kg) twice per week. As shown in Fig. 6A–C, abiraterone feeding alone had no effect on tumour size and weight; however, KAT2A knockdown in combination with abiraterone treatment decreased the volume and weight of the tumour, suggesting that KAT2A knockdown might reverse abiraterone resistance in CRPC. The acetylation level in xenograft tumour tissues was also decreased in the KAT2A-knockdown group (Fig. 6D). In addition, IHC showed that the levels of KAT2A protein decreased significantly in stably knocked down C4-2-AbiR cells in vivo. AR was mostly distributed in the nucleus in C4-2-AbiR cells and was scattered within the cytoplasm upon KAT2A knockdown, but without an evident decline in protein levels between treatment groups. Moreover, the combination of KAT2A knockdown and abiraterone resulted in weaker staining intensities of PSA and Ki67 compared to those in the KAT2A knockdown-only group, indicating that the deletion of KAT2A enhanced the inhibitory effect of abiraterone on AR activity and the proliferation of C4-2-AbiR tumours (Fig. 6E). Taken together, these results suggested that KAT2A knockdown efficiently reversed hormonal therapy resistance in CRPC, highlighting KAT2A as a novel therapeutic target for abiraterone-resistant CRPC.

## Discussion

HATs, also known as lysine acetyltransferases (KATs), are a group of enzymes that can directly acetylate histone lysine residues to modify transcriptional function by weakening the interaction between histones and DNA and recruit DNA-binding proteins, which serve as docking sites [44, 45]. Several recent studies have shown that KATs target other substrates along with histones, indicating that non-histone proteins can also be acetylated by KATs to regulate cell functions such as protein folding, RNA stability, and autophagy [46]. KAT2A, a member of the GCN5-related N-acetyltransferase (GNAT) family of KATs, has been reported to be abnormally expressed and to play an essential role in the progression of various types of cancer [30, 34, 35]. However, the expression and function of KAT2A in PC have not been elucidated thus far. In this study, we have shown that KAT2A expression is elevated in PC tissues compared with that in adjacent normal tissues. Additionally, the up-regulated expression of KAT2A was significantly correlated with poor clinical outcomes or high-risk factors such as overall survival, PFS, and higher a Gleason score, which suggested that KAT2A plays a critical role in the progression of PC and might act as a potential prognostic indicator for PC patients.

In hormone-sensitive PC, the cancer cells rely on androgen binding to the AR to induce tumourigenesis [47]. Even after androgen deprivation treatment, which is aimed at reducing androgen synthesis, the activation of AR-dependent pathways continues to play an important role in the progression of CRPC [5]. The mechanisms underlying AR signalling activation include the unregulated expression of androgen synthesis-related genes, intra-tumour elevated androgen, AR variants, AR gene mutation, abnormal expression of AR, and PTM [48]. Abiraterone, an inhibitor of CYP17A that is mainly involved in androgen synthesis, has been demonstrated to significantly prolong the survival of CRPC patients [49]. However, CRPC patients tend to exhibit primary and acquired resistance to abiraterone; although the underlying resistance mechanisms remain unclear, they have been suggested to be linked to alterations in AR and other signalling pathways [9–11]. The abiraterone-resistant cell line C4-2-AbiR we established showed slower growth rate, which might be induced for adapting to the stress of drug in circumstance [50]. And we also found that the protein level of KAT2A was increased in C4-2-AbiR. Knocking down KAT2A could recover the inhibitory effect of abiraterone on C4-2-AbiR cell proliferation, whereas KAT2A overexpression promoted resistance to abiraterone in LNCaP-P and C4-2-P cells. These results strongly indicate that KAT2A might facilitate resistance to abiraterone in PC cells.

Activation of the AR signalling pathway is significant for the progression of CRPC [5]. Translocation of AR to the nucleus is necessary for binding to androgen response elements (AREs) to enable its transcriptional function [48]. In our study, it seemed that R1881, a kind of AR agonist, could not trigger AR translocation into the nucleus obviously in C4-2-AbiR, whereas it could do this in LNCaP-P cells. This was probably because C4-2-AbiR were exogenous androgen independent and had lost their androgen responsiveness so that no apparent effect of R1881 was noticed, which was in accordance with the research of H C Wu et al who constructed the C4-2 cell line originally [51] and other researches [52]. Besides, FBS-depleted condition was prone to block the translocation of AR into nucleus to some extent, and the effect in LNCaP was more obvious than that in C4-2 cells, which might due to C4-2 had the stronger cell viability when cultured in serum-free medium consistent with the existing studies [19, 51].

Studies have shown that the nuclear translocation of AR is mediated by a bipartite nuclear localisation signal located within the hinge region, suggesting that the hinge region of AR has a strong correlation with its translocation into the nucleus [53, 54]. Three lysine residues, K630, K632, and K633, located in the hinge region can be acetylated by several KATs, resulting in the translocation of AR into the nucleus [18, 19, 55]. Mutation of these residues to alanine results in the absence of acetylation on AR [18]. Our study showed that up-regulation of KAT2A could drive AR binding with AREs within the PSA promoter and could increase the expression of PSA, in addition to regulating the expression of AR. Interestingly, KAT2A initiated AR translocation into the nucleus, as detected via immunofluorescence and nuclear extraction. Considering the non-histone acetylation of KATs and potential acetylated sites within the hinge region of AR, we confirmed that AR could be acetylated by KAT2A at the K630 residue, leading to its translocation into the nucleus. Mutation of K630A blocked the KAT2A-induced acetylation of AR, whereas mutation of K630Q tended to promote AR translocation into the nucleus even without KAT2A expression.

In summary, the present data showed that KAT2A promotes resistance to abiraterone and that targeting KAT2A might reverse abiraterone therapy resistance in CRPC. The combination of targeting KAT2A with abiraterone treatment might provide a new therapeutic strategy for CRPC.

## Supplementary information

Supplementary information

## Data Availability

The datasets used and/or analyzed during the current study are available from the corresponding author on reasonable request.
