# Supplementary material for: KAT2A-mediated AR translocation into nucleus promotes abiraterone-resistance in castration-resistant prostate cancer
Source: Cell Death Dis. 2021 Aug 12;12(8):787. doi: 10.1038/s41419-021-04077-w (PMC8357915; doi:10.1038/s41419-021-04077-w)
Supplement: Supplementary file 1 — Supplementary information [file 41419_2021_4077_MOESM1_ESM.docx]

**Supplementary Figure S1**

**
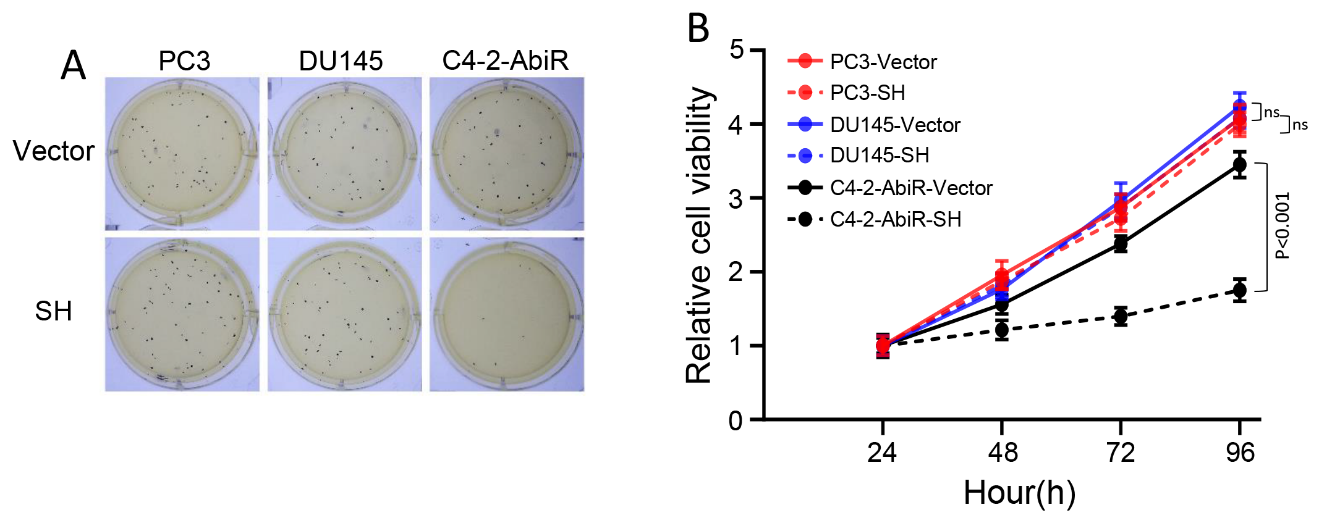
**

**Supplementary Figure S1.** The effect of abiraterone on AR negative cell lines and AR positive cell line transfected with KAT2A-silencing plasmids or vectors. **a** Proliferation ability of AR negative PC cell lines PC-3 and DU145 and AR positive C4-2-AbiR with sh-KAT2A or vectors under abiraterone circumstance tested by soft agar assay. **b** Relative cell viability of PC-3, DU145 and C4-2-AbiR with sh-KAT2A or vectors under abiraterone circumstance tested by CCK-8.

**Supplementary Figure S2**


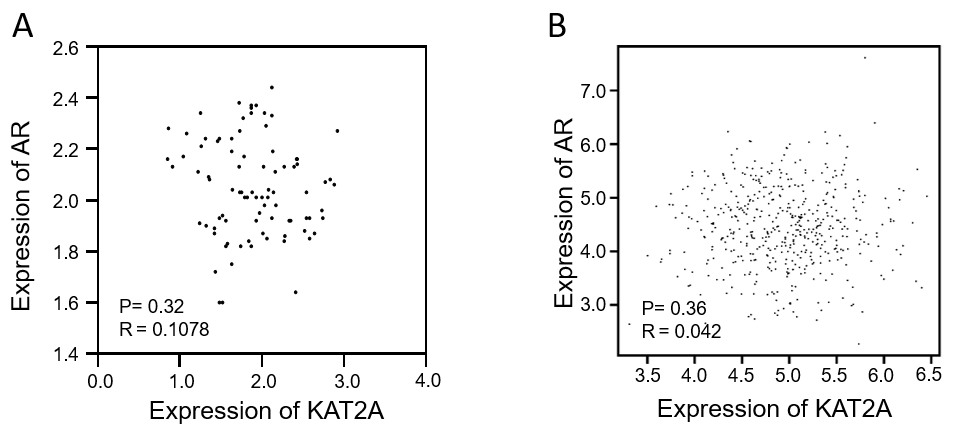


**Supplementary Figure S2.** The correlation between expression of KAT2A and AR. **a** The correlation between KAT2A and AR expression based on IHC H-score of 87 samples. **b** The correlation between KAT2A and AR expression based on TCGA database.

**Supplementary Figure S3**


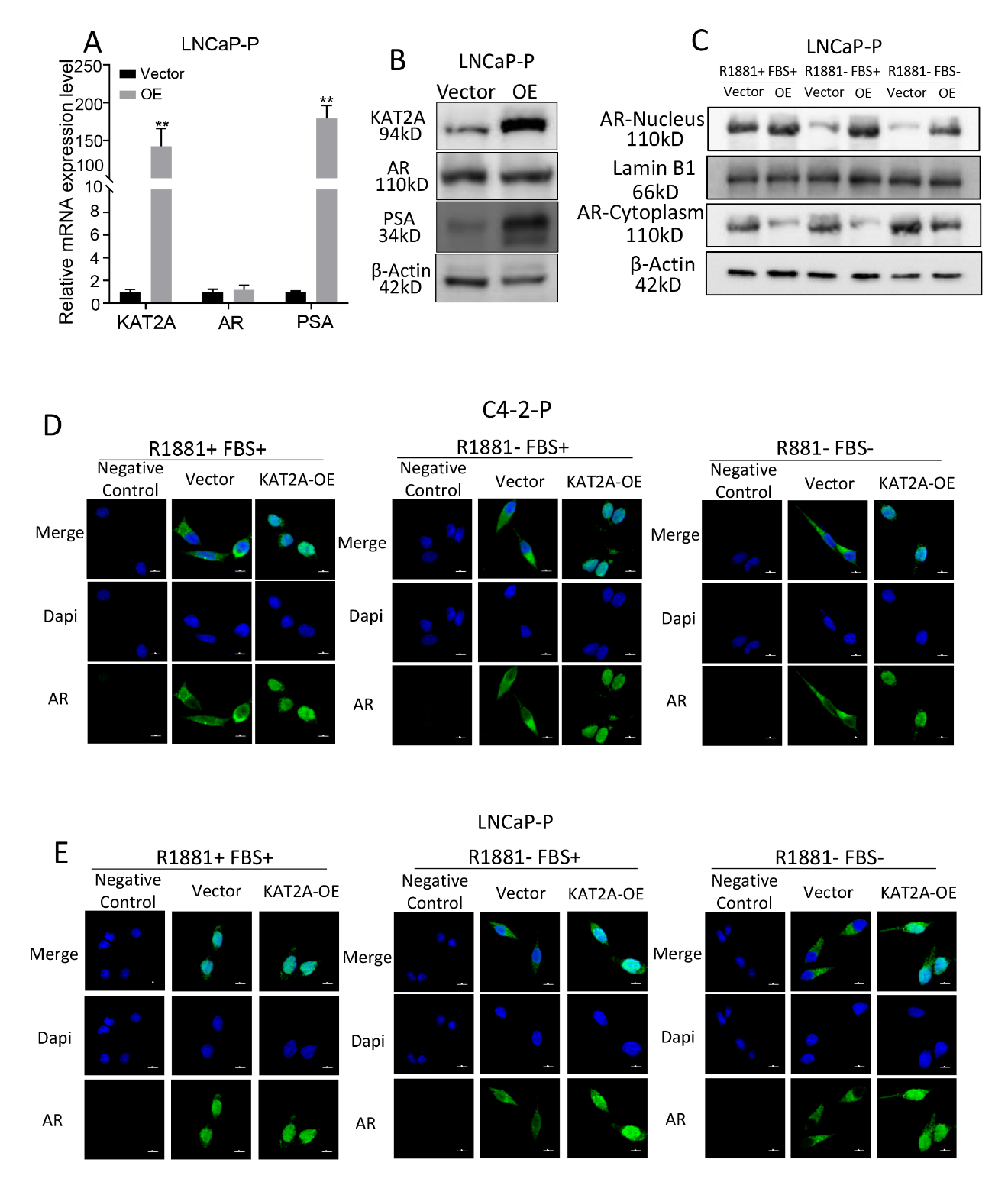


**Supplementary Figure S3.** Resistance to abiraterone can be modified by upregulation of KAT2A in parental PC cell line. **a** Relative mRNA expression levels of KAT2A, AR and PSA in LNCaP-P stably transfected with OE-KAT2A or vectors were detected by qRT-PCR. **b** The protein levels of KAT2A, AR and PSA in LNCaP-P stably transfected with OE-KAT2A or vectors were detected by western blotting. **c** Aggregates of AR in cytoplasm or nucleus in LNCaP-P stably transfected with oe-KAT2A or vectors were extracted and detected. **d** Location of AR in C4-2-P stably transfected with oe-KAT2A or vectors was detected by IF. **e** Location of AR in LNCaP-P stably transfected with oe-KAT2A or vectors was detected by IF. **p* < 0.05, ***p* < 0.01

**Supplementary Figure S4**

**
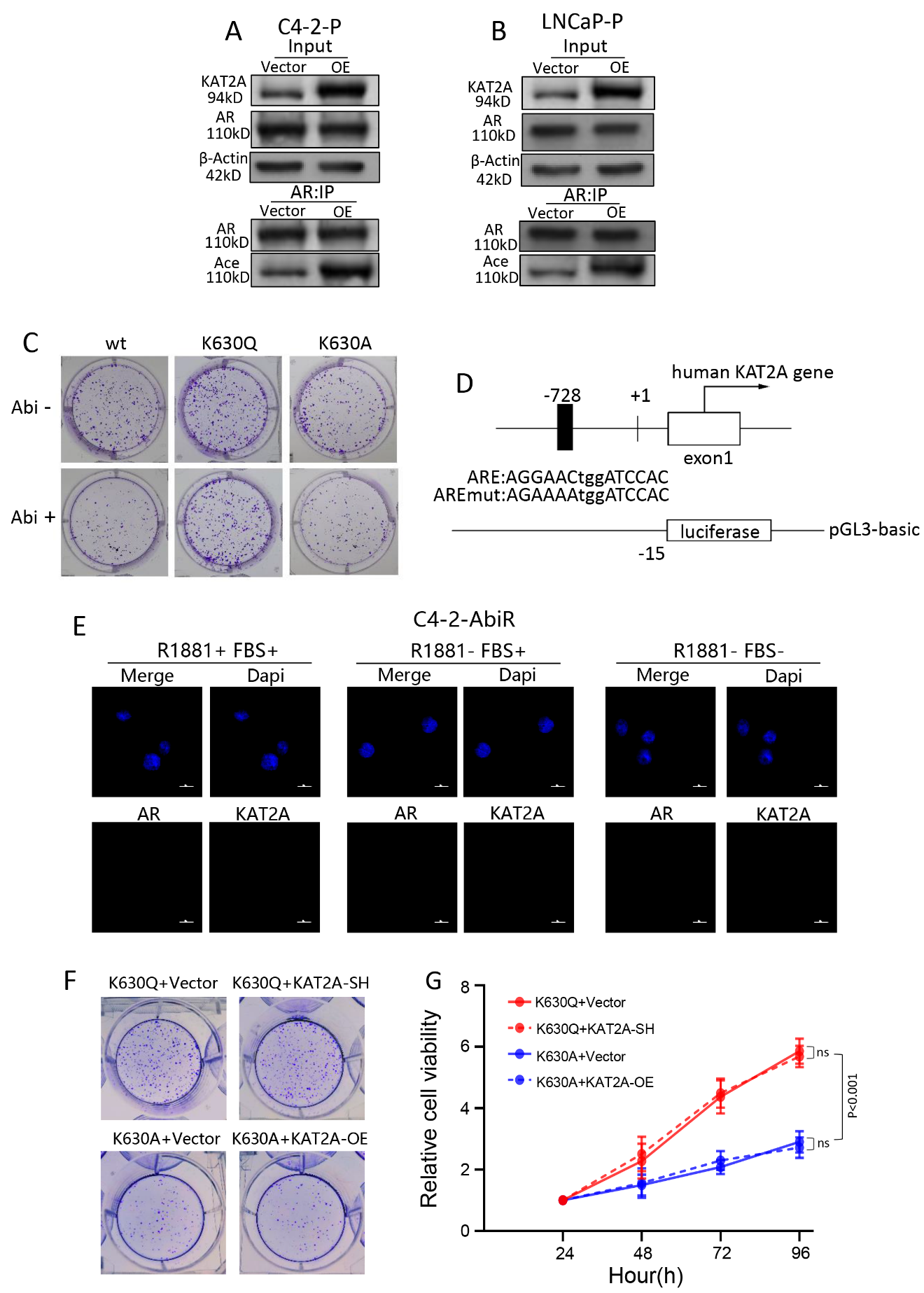
**

**Supplementary Figure S4. a, b** Acetylation level of AR in C4-2-P and LNCaP-P stably transfected with oe-KAT2A detected by pan-acetylation antibody after immunoprecipitation by AR antibody from cell lysates. **c** Proliferation of PC-3 cell line stably transfected with AR of wild type, K630Q or K630A with or without abiraterone. **d** Pattern of pGL3-basic containing promoter of KAT2A and mutant. **e** The negative control of IF in testing co-localisation of KAT2A and AR in C4-2-AbiR. **f** Proliferation of PC-3 cell line stably transfected with K630Q/K630A and KAT2A-SH/ KAT2A-OE/ vector with abiraterone tested by colony formation assay. **g** Relative cell viability of PC-3 cell line stably transfected with K630Q/K630A and KAT2A-SH/ KAT2A-OE/ vector with abiraterone tested by CCK-8.

**Supplementary Table 1: qRT-PCR primer sequences in this study**

| Gene names | Primer sequence |
| --- | --- |
| GAPDH-F | 5′-GGTCGGAGTCAACGGATTTG-3′ |
| GAPDH-R | 5′-GGAAGATGGTGATGGGATTTC-3′ |
| KAT2A-F | 5′-CTCTGCCTTAACTACTGGAAGC-3′ |
| KAT2A-R | 5′-GCCATCTGGTGTAATTGACCTTG-3′ |
| AR-F | 5′-ACTTTGAGGCTGTCAGAGCG-3′ |
| AR-R | 5′-AGGCTGTGATGATGCGGTAG-3′ |
| PSA-F | 5′-GATGCTGTGAAGGTCATGGA-3′ |
| PSA-R | 5′-TGGAGGTCCACACACTGAAG-3′ |

**Supplementary Table 2: KAT2A-sh sequences in this study**

| Name | Sequence |
| --- | --- |
| KAT2A-sh1 | 5′-CCGGGCGCATGCCTAAGGAGTATATCTCGAGATATACTCCTTAGGCATGCGCTTTTTG-3′ |
| KAT2A-sh2 | 5′-CCGGGGCTACCTACAAGGTCAATTACTCGAGTAATTGACCTTGTAGGTAGCCTTTTTG-3′ |

**Supplementary Table 3: Drugs and reagents**

| Drug / Reagent | Source | Identifier / formulation |
| --- | --- | --- |
| PI/RNase Staining Buffer | BD Biosciences | Catalog No: 550825 |
| Abiraterone acetate | MedChemExpress | Catalog No: HY-75054 |
| R1881 | Sigma | Catalog No: R0908 |
| Deacetylase Inhibitor Cocktail | MedChemExpress | Catalog No: HY-K0030 |

**Supplementary Table 4： Primary and secondary antibodies used in this study**

| Antibody | Source | Identifier | Host |
| --- | --- | --- | --- |
| KAT2A antibody | CST | Catalog No: 3305T | Rabbit |
| KAT2A antibody | Proteintech | Catalog No: 66575-1-Ig | Mouse |
| AR antibody | CST | Catalog No: 5153T | Rabbit |
| Acetylated-Lysine antibody | CST | Catalog No: 9441S | Mouse |
| PSA antibody | Proteintech | Catalog No: 10679-1-AP | Rabbit |
| Lamin B1 antibody | Proteintech | Catalog No: 12987-1-AP | Rabbit |
| β-actin antibody | Proteintech | Catalog No: 66009-1-Ig | Mouse |
| His-Tag antibody | Proteintech | Catalog No: 10001-0-AP | Rabbit |
| Flag-Tag antibody | Proteintech | Catalog No: 20543-1-AP | Rabbit |
| HRP conjugated Goat anti-mouse antibody | Proteintech | Catalog No: SA00001-1 | Goat |
| HRP conjugated Goat anti-rabbit antibody | Proteintech | Catalog No: SA00001-2 | Goat |
